# Pharmacogenomics: An Update on Biologics and Small-Molecule Drugs in the Treatment of Psoriasis

**DOI:** 10.3390/genes12091398

**Published:** 2021-09-10

**Authors:** Valerio Caputo, Claudia Strafella, Terenzio Cosio, Caterina Lanna, Elena Campione, Giuseppe Novelli, Emiliano Giardina, Raffaella Cascella

**Affiliations:** 1 Medical Genetics Laboratory, Department of Biomedicine and Prevention, Tor Vergata University, 00133 Rome, Italy; v.caputo91@gmail.com (V.C.); claudia.strafella@gmail.com (C.S.); novelli@med.uniroma2.it (G.N.); emiliano.giardina@uniroma2.it (E.G.); 2 Genomic Medicine Laboratory UILDM, IRCCS Santa Lucia Foundation, 00179 Rome, Italy; 3 Dermatologic Unit, Department of Systems Medicine, Tor Vergata University, 00133 Rome, Italy; terenziocosio@gmail.com (T.C.); caterinalanna.cl@gmail.com (C.L.); campioneelena@hotmail.com (E.C.); 4 IRCCS Neuromed, 86077 Pozzilli, Italy; 5 Department of Pharmacology, School of Medicine, University of Nevada, Reno, NV 89557, USA; 6 Department of Biomedical Sciences, Catholic University Our Lady of Good Counsel, 1000 Tirana, Albania

**Keywords:** psoriasis, psoriatic arthritis, biomarkers, pharmacogenomics, polymorphisms, drug, treatment

## Abstract

Pharmacogenomic studies allowed the reasons behind the different responses to treatments to be understood. Its clinical utility, in fact, is demonstrated by the reduction in adverse drug reaction incidence and the improvement of drug efficacy. Pharmacogenomics is an important tool that is able to improve the drug therapy of different disorders. In particular, this review will highlight the current pharmacogenomics knowledge about biologics and small-molecule treatments for psoriasis. To date, studies performed on genes involved in the metabolism of biological drugs (tumor necrosis factor inhibitors and cytokines inhibitors) and small molecules (apremilast, dimethyl fumarate, and tofacitinib) have provided conflicting results, and further investigations are necessary in order to establish a set of biomarkers to be introduced into clinical practice.

## 1. Introduction

The transition from reactive to proactive care has outlined new management strategies, which are oriented towards the maintenance of patient welfare. This healthcare approach includes the exploration of the human genome, with the aim of identifying different genetic variants (single-nucleotide variants, SNVs; copy number variations, CNVs; small insertions and deletions, INDELs) that are involved in diseases susceptibility, drug response degree, and adverse drug reactions (ADRs). In particular, the collection of these data can be employed for the development of personalized treatments, designed in relation to the patient’s genomic profile. To this purpose, pharmacogenomics is one of the personalized medicine tools, which plays an important role in the management of therapeutic treatments [1]. On the other hand, the implementation of pharmacogenomics is encouraged by the identification and validation of specific biomarkers that can be detected in different biological sources (blood, saliva, tissue). SNVs, CNVs, and INDELs can be recognized as pharmacogenomic biomarkers that are able to affect the efficacy and toxicity of drugs [2,3]. Moreover, pharmacogenomic biomarkers were utilized to develop several genetic tests, with the aim of predicting the response degree or ADRs to specific treatments. Their clinical utility has been widely shown by the reduction in ADR incidence and the improvement of drug efficacy [4,5]. To date, pharmacogenomics can be considered as a tool to improve the drug therapy of several diseases or viral infections, such as inflammatory bowel disease (IBD), cardiovascular disease (CD), cancer, psoriasis (Ps), hepatitis C virus (HCV), and human immunodeficiency virus (HIV) [6,7,8,9,10,11].

In particular, Ps is a chronic immune-mediated inflammatory skin disease, with a world-wide prevalence of 2–3% according to the different ethnicities and geographical regions [12]. It is possible to distinguish several phenotypes of Ps, depending on the appearance of skin lesions. Indeed, cutaneous manifestations are numerous, with a vulgar form that is characterized by well-circumscribed erythematous plaques, covered by a squamous scale, preferentially located on the skin of extensor surfaces of the body [13]. The most common form is plaque Ps that affect approximately 90% of patients. Furthermore, approximately 20–30% of Ps patients develop psoriatic arthritis (PsA) during their lifetime [14].

Numerous comorbidities (Crohn’s disease, psychological/psychiatric disorders, and uveitis) have been associated with Ps, although metabolic syndrome (MS) represents the most common one. In fact, psoriatic subjects have an increased risk of developing cardio-metabolic diseases (CVD) underling the systemic features of Ps [15,16].

Ps severity is classified as mild, moderate, and severe, which is determined by the body surface area (BSA), psoriasis area and severity index (PASI), physician global assessment (PGA), and dermatology life quality index (DLQI) [16]. Ps is described as a multifactorial disorder, in which environmental and genetic/epigenetic factors are likely to influence the susceptibility to the disease. To date, the genetic contribution has been investigated, highlighting the existence of a large number of risk variants associated with the onset and/or progression of Ps [17,18,19].

Currently, genomic and epigenomic studies are useful tools to detect novel targets for Ps therapeutic options. Concerning Ps therapeutic approaches, nowadays there are numerous available treatments to mitigate symptoms, clinical manifestations, and to manage comorbidities. In fact, treatment selection needs to take into account the presence of comorbidities associated with the disease [20]. In this regard, it is necessary to search specific drugs that are able to reduce ADR, whilst maintaining pleiotropic capacity. Moreover, correct management of the disease also requires the ability to distinguish between primary and secondary non-response. In particular, primary non-response is usually considered if the drug results to be ineffective (i.e., no clinical response within the initial treatment period). On the other hand, a non-secondary response can occur when, after the first response to the treatment, the effectiveness of the drug is lost over time [21]. The cause of non-responder patients could be related to the drug, to the treatment regimen, or to the patient genomic/epigenomic background. Moreover, non-responder patients could also develop an immunogenicity to biological drugs, and also to biosimilars. Since biologics originate from fusion proteins, antibodies, or parts of antibodies, they can induce the development of anti-drug antibodies (ADAs), a phenomenon known as drug immunogenicity. In fact, ADAs may be considered as the cause of the initial non-response or reduced therapeutic effect over the course of the disease. Although there are obvious differences between biologics, in terms of ADA, there are no accepted dermatological guidelines or drug classes for preventing the ADA risk. Furthermore, there is no consensus on the method for detecting ADA. Therefore, better characterization of patients at risk of developing ADA is needed to establish the clinical decision making [22].

The aim of this review is to update the state of art of Ps pharmacogenomic studies focusing on biologics and small-molecule treatments, taking into account the scientific literature published from 2020 to 2021.

## 2. Materials and Methods

This review was written through the collection of scientific data from the following databases: Cochrane Central Register of Controlled Trials, MEDLINE, Embase, US National Institutes of Health Ongoing Trials Register, NIHR Clinical Research Network Portfolio Database, World Health Organization International Clinical Trials Registry Platform, CDC (Centers for Disease Control and Prevention), CPIC (Clinical Pharmacogenetics Implementation Consortium), GPA (Global Psoriasis Atlas) and PubMed. Reference lists and scientific articles were published from January 2020 until May 2021. A research was performed including the following different keywords: “psoriasis”, “psoriasis treatment”, “pharmacogenetics”, “pharmacogenetic variants”, “single-nucleotide polymorphism” or “SNPs”, “biological drugs”, “small molecule treatment”, “apremilast”, “dimethyl fumarate”, “tofacitinib”, “brodalumab”, “guselkumab”, “ixekizumab”, “secukinumab”, “adalimumab”, “etanercept”, “infliximab”, “certolizumab”. Only peer-reviewed international studies were included in the research and it was restricted to human studies, with no restrictions of age, sex, ethnicity or type of study. Case reports and case series were included if they described data not present in reviews or clinical trials. Phase I clinical trials have not been included in this review. Initially, 17 papers matched with our search criteria and subsequently, 11 scientific articles were excluded. Therefore, the final evaluation was performed on 6 articles.

## 3. Pharmacogenetics of Ps Treatments

### 3.1. Biological Drugs

Biopharmaceutical or biologics are drugs derived from biological sources (humans, animals, plants, microorganisms), which act on specific molecular pathways, in order to prevent or treat immune-mediated inflammatory disorders. The biologics utilized to treat Ps can be classified in two different groups, according to the therapeutic target, such as tumor necrosis factor (TNF) inhibitors and IL-23/*IL-17* inhibitors [23,24].

#### 3.1.1. Pharmacogenetics of Anti-Tumor Necrosis Factor Drugs

TNF plays a central role in the activation of several signaling pathways leading to the stimulation of cells involved in the inflammatory and immune response. In particular, TNF engages macrophages, T-cells, B-cells, pro-inflammatory cytokines (IL-1, *IL-6*), and chemokines (IL-8, RANTES). The *TNF* or *TNF-α* gene is located on the short arm of chromosome 6 (6p21.33), and encodes for a multifunctional pro-inflammatory cytokine as a member of the TNF superfamily. This cytokine, mainly secreted by macrophages, is involved in the regulation of a wide spectrum of biological processes, such as cell proliferation, differentiation, and lipid metabolism. Moreover, *TNF* is involved in the production of T-cells, which trigger the local proliferation of epidermal keratinocytes and, thereby, plays an essential role in the etiopathogenesis of psoriatic lesions [25].

Anti-TNF drugs are one of the treatment options for psoriatic patients, which are resistant to conventional systemic therapies. These therapeutic approaches have also shown good efficacy, even if 30–50% of psoriatic patients do not show enhancement of their health status [26]. Anti-TNF therapy may be responsible for severe adverse events, causing paradoxical psoriasiform reactions and changing the morphology of psoriatic lesions [27]. In addition, *TNF-α* inhibitors are used to treat patients affected by IBD. As reported, these patients have an increased risk of developing ADRs, due to overproduction of interferon-α by plasmacytoid dendritic cells, resulting in the activation and amplification of pathogenic T-cells [28].

The *TNF-α* inhibitors employed in Ps treatments are infliximab (INF), adalimumab (ADA), certolizumab (CTL), and etanercept (ETN). INF, ADA, and CTL are monoclonal antibodies against *TNF-α*, whereas ETN is a recombinant fusion protein [29]. ADA, INF, and CTL are indicated for the treatment of moderate-to-severe Ps. The efficacy of these monoclonal antibodies is influenced by different genetic variants located on the *TNF-α* gene [30,31]. To date, more than 200 genetic variants have been identified. In particular, the polymorphisms *TNF-308* (A/G, rs1800629), *TNF-238* (G/A, rs361525), and *TNF-857* (C/T, rs1799724) have been extensively investigated, either as risk or pharmacogenetic biomarkers [31]. The role of these SNPs has been evaluated in a meta-analysis that examined psoriatic patients treated with ETN, ADA, and INF, showing controversial results [32]. In fact, the G allele of rs1800629 and the G allele of rs361525 were significantly associated (*p* = 0.000086 and *p* = 0.016, respectively) with a good response to anti-TNF drugs in European patients. However, the association of these genetic variants failed to be significant in the Asian cases. Furthermore, studies performed on rs1799724 reported that only European patients carrying the C allele showed a better response. Another genotyping study revealed that *TNF-1031* (T/C, rs1799964), located on the promoting region of the *TNF* gene, is associated with a good response to anti-TNF drugs. In particular, patients with the TT genotype and treated with INF showed the highest response at 3 months and at 6 months after treatment. Moreover, the bioinformatic analysis reported that rs361525 and rs1800629 modify regulatory regions linked to specific biological process, such as epithelial–mesenchymal transition (EMT). EMT is involved in cell morphology changes. In fact, this mechanism allows a polarized epithelial cell to assume a mesenchymal cell phenotype, increasing its migratory capacity, invasiveness, and high resistance to apoptosis. This result suggests that EMT could be involved in epithelium changes in patients affected by Ps [33].

The metabolism of anti-TNF drugs can also be influenced by the *TNFAIP3* gene (6q23.3). In particular, pharmacogenomic studies revealed the existence of two polymorphisms (rs610604, G/A/C/T and rs6920220, G/A) in *TNFAIP3*, which may be potentially involved in the different outcomes of treatments. However, the association studies reported conflicting results. In fact, the lack of association (*p* > 0.05) was described in Ps patients treated with ADA or ETN, whereas, the rs610604 and rs6920220 were associated (*p* = 0.041 and *p* = 0.025, respectively) with a good response to anti-TNF drugs [29,31].

The SNP rs2546890 (A/G) is located in the *IL-12B* gene (5q33.3), and may influence the response to treatment with ETN, ADA, and INF. In particular, the presence of the G allele could be associated with a worse response. In addition, polymorphisms (rs1143623, C/A/G; rs1143627, G/A) of *IL-1B* (2q14.1) may affect the response to anti-TNF drugs. A research study revealed that psoriatic patients carrying the GG genotype of rs1143623 and the AA genotype of rs1143627 presented a worse response if treated with anti-TNF drugs. Another gene involved in the metabolism of anti-TNF drugs is *IL-6* (7p15.3). The GG genotype of SNP rs1800795 (C/G/T) may influence the response to anti-TNF drugs, showing a worse response [29,31,33].

The genotype CT of rs763780 (C/T), located in the *IL-17A* gene (6p12.2), indicated a good response in patients subjected to INF treatment.

The rs4819554 (G/A/C), located in the *IL-17-RA* gene (22q11.1), could play a role in the response to anti-TNF drugs. In fact, psoriatic patients carrying the A allele showed a better response to anti-TNF treatment.

Another IL potentially involved in the pharmacogenomics of Ps is *IL-23R*, encoded by the *IL-23R* gene located on the 1p31.3 locus. A research study, performed on psoriatic Spanish patients, revealed that the GG genotype of rs11209026 (A/G) was associated with a good response to the treatment. Moreover, the rs11209026 could be associated with the risk of developing ADR [29,31,33].

The *human leukocyte antigen* (*HLA*) system is located on the short arm of chromosome 6 (6p21) and is clinically relevant as a transplantation antigen, playing a crucial role in the regulation of the immune response. The *HLA* polymorphisms have been described as risk and pharmacogenetic biomarkers. In particular, rs13437088 (C/T, *HLA-B*) was associated with the response to ETN, while rs1048554 (G/A/C, *HLA-C*) showed a good response to anti-TNF drugs. The presence of the HLA-Cw*06 allele may be associated with a lower probability of responding to ADA, ETN, or INF [29,31].

The genome-wide association study (GWAS) reported new potential candidate genes (*AKAP13*, *HNRNPKP3*, *SUPT3H*, *NPFFR2,* and *CDH12*) involved in the metabolism of anti-TNF drugs. In Table 1 are the summarized genes and polymorphisms that might have a potential impact on the response to anti-TNF drugs (Table 1).

It is important to remark that most of the pharmacogenomic biomarkers previously described have not been confirmed in a different cohort [34]. As reported in this paragraph, several studies have been performed, in order to identify pharmacogenomic biomarkers that can be introduced into clinical practice. However, most of these biomarkers have not been validated and, consequently, cannot be used as a supporting tool for the management of anti-TNF drug therapy.

#### 3.1.2. Pharmacogenetics of Cytokine Inhibitors

Cytokines are water-soluble extracellular polypeptides produced by different types of cells. The cytokine-targeted therapy for the treatment of Ps patients involves the administration of *IL-17* inhibitors (secukinumab, SCK; ixekizumab, IXE; brodalumab, BDL) targeting the *IL-17RA* receptor. Other cytokine inhibitors utilized for the treatment of Ps are guselkumab (GSL), tildrakizumab (TDK), risankizumab (RSK), and ustekinumab (UTK). In particular, GSL, TDK, and RSK inhibit IL-23, or its receptor *IL-23R*. On the other hand, UTK is able to block the biological activities of IL-12. Furthermore, these drugs have a different effect on the outcomes in moderate-to-severe phenotypes, and this condition may be explained by the existence of genetic polymorphisms linked to the individual variability to a drug response. To this purpose, it has been estimated that 50–70% of psoriatic patients show different response degrees to anti-ILs drugs [35].

The *IL-17A* gene is a member of the *IL-17* receptor family and encodes a pro-inflammatory cytokine. *IL-17A* is involved in the production of inflammatory molecules, chemokines, antimicrobial peptides, and remodeling proteins. Moreover, *IL-17* has an impact on host defense, cell trafficking, immune modulation, and tissue repair, playing a crucial role in the activation of innate immune defenses. As extensively demonstrated, high levels of *IL-17* are associated with several chronic inflammatory diseases, including rheumatoid arthritis, Ps, and multiple sclerosis [36]. *IL-17* polymorphisms are associated with different response degrees to cytokine inhibitors. In particular, the heterozygous genotype CT of rs763780 (C/T) was correlated to a worse response to UTK [29,31]. A recent study described six SNPs that could alter the molecular processes (hyper-proliferation of keratinocytes, T-cell polarization, and EMT) involved in the pathophysiology of Ps [33]. *IL-17RA* (22q11.1) is mainly involved in the keratinocytes hyper-proliferation process, and the SNP rs4819958 (G/A) may modify the interaction with *PAX1* and *NR2F1*.

SCK and IXE are fully human monoclonal antibodies that target *IL-17A*. IXE also binds to the heterodimer form of the protein (IL-17A/F). These drugs provide a fast initial response that is maintained over time, with high safety profile [37,38]. Moreover, due to the increased risk of fungal infection in *IL-17* inhibitor-treated patients, a screening of the *CLEC7A* (12p13.2) gene should be included in the clinical algorithm, to stratify the target population. This gene encodes for Dectin-1, which is a natural killer (NK) cell receptor-like C-type lectin that is thought to be involved in innate immune responses to fungal pathogens. In particular, genetic variants in Dectin-1 could predispose to fungal infection, and concomitant use of the *IL-17* inhibitor could increase this risk [39,40].

The majority of pharmacogenetic studies are focused on SCK [31,41]. Preliminary data reported that the presence of the HLA-C*06:02 allele may influence the effectiveness and safety of SCK [42]. However, this result was not confirmed, as SCK proved to be effective independently of the *HLA-C* genotype [43,44]. The research of pharmacogenetic biomarkers was performed in a multicenter study, focusing on *IL-17A* inhibitors (SCK and IXE). In particular, they analyzed the coding regions and untranslated regions (UTR) of *IL-17A*, identifying five SNPs (rs3748067, C/T; rs2275913, C/G/T; rs3819025, A/G; rs7747909, A/G and rs8193037, A/C/T) located in non-coding regions. As reported, these genetic variants did not act on the efficacy of these drugs after 12 weeks of treatment [45].

A recent study reported that SNPs at the *HLA-C* locus did not influence the response to SCK. On the other hand, rs9267325 (G/C, *MICB-DT*, 6p21.33), rs34085293 (T/G, *DDX58*, 9p21.1), and rs2304255 (C/T, *TYK2*, 19p13.2) have been associated with a good response to SCK. The HLA-Cw6 and other *HLA-C* alleles, as well as *MICB-DT*, *DDX58*, and *TYK2* genetic variants, have been correlated with an optimal response to anti-*IL-17A* treatment. These results suggest the possibility to create a panel of SNPs to be used for optimizing the use of anti-*IL-17A* treatment [29,31]. Furthermore, *IL-17RA* could play an important role in the metabolism of BDL [33]. BDL is a human monoclonal immunoglobulin G2 antibody that selectively binds to human *IL-17RA*, thereby inhibiting its interaction with other cytokines, such as *IL-17A*, IL-17F, IL-17C, IL-17A/F heterodimers, and IL-25. In particular, BDL blocks IL-17RA activity and, consequently, inhibits *IL-17*-induced responses, including the release of pro-inflammatory cytokines and chemokines implicated in the development of plaque Ps [46]. Moreover, a research study assessed the efficacy of BDL in psoriatic patients who failed the previous treatment with *IL-17A* inhibitors (SCK and IXE). The evaluation of PASI demonstrated the major efficacy of BDL. These data suggest the possibility of proposing an alternative therapy with BDL to patients who do not respond to SCK and IXE treatments [46,47].

Anti-IL-12 and *IL-23* are monoclonal antibodies as UTK, RSK, and GSL, respectively. The *IL-12B* gene encodes for the p40 subunit of IL12, which acts on T and natural killer cells. *IL-12* links, in turn, with *IL23A* to create IL-23. Polymorphisms (rs2546890, A/G; rs3213094, C/G/T) located in *IL-12B* may alter the response to UTK, an IL-12/23 inhibitor. In fact, patients with the CT genotype for the SNP rs3213094 showed a better response to UTK. Recent meta-analysis studies revealed that RSK and GSL are the most effective drugs at 12–16 weeks of treatment. Moreover, UTK showed lower efficacy than RSK and GSL also depending on the dosage.

SNPs (rs1143623, C/A/G and rs1143627, G/A) located in *IL-1B* may alter the response to UTK treatment. In fact, the GG genotype of rs1143623, or AA genotype of rs1143627 are associated with a worse response [29,31].

Another gene potentially involved in the metabolism of UTK is the *TNFAIP3* gene. Several studies failed to highlight any significant association (*p* > 0.05) between rs610604 (G/A/C/T) and the response to UTK. Afterwards, a study performed on psoriatic patients from the Netherlands reported that the GG genotype of rs610604 is associated (*p* = 0.031) with a worse response to UTK [29,31].

Moreover, new potential biomarkers (*TLRs*, *PGLYRP4*, *FBXL19*, *CARD14*) have been investigated, although they need to be confirmed by further studies [31]. In Table 2 are the summarized genes and polymorphisms that might have a potential impact on the response of cytokine inhibitors (Table 2). In fact, it will be necessary to perform studies on a larger cohort of patients, replicate them in different populations, and, possibly, identify population-specific biomarkers.

### 3.2. Small Molecules

Small-molecule inhibitors (SMIs) are new-generation drugs that find an excellent use, as they can easily enter cells thanks to their low molecular weight. In particular, treatments for Ps include the administration of oral SMIs, such as apremilast, dimethyl fumarate, and tofacitinib [45].

#### 3.2.1. Phosphodiesterase-4 Inhibitor

Phosphodiesterases (PDEs) represent a large family of enzymes that regulate the intracellular levels of cyclic nucleotides. PDEs are able to terminate cyclic nucleotide signaling by catalyzing the hydrolysis of cAMP and cGMP. In particular, phosphodiesterase-4 (PDE-4) regulates the inflammatory response by degrading cAMP [18]. The cAMP-specific PDE-4 is highly expressed in the brain, cardiovascular tissues, smooth muscles, keratinocytes, and immune cells (T cells, monocytes, macrophages, neutrophils, dendritic cells, eosinophils) [48]. In the skin, PDE-4 is principally expressed in keratinocytes, neutrophils, Langerhans, and T cells, which contribute to the formation of the psoriatic plaque. The inhibition of PDE-4 can increase the intracellular level of cAMP, and subsequently modulate the inflammatory responses and maintain the immune system homeostasis [49]. Different PDEs (roflumilast, apremilast, and crisaborole) were approved for the treatment of inflammatory disorders or skin diseases.

Apremilast has been licensed by the US Food and Drug Administration (FDA) for the management of active PsA (21 March 2014) and moderate-to-severe plaque Ps (23 September 2014). Apremilast acts significantly on the inhibition of inflammatory responses. In fact, this small molecule reduces circulating levels of Th1 and Th17 pro-inflammatory mediators, and increases anti-inflammatory mediators. PDE-4 inhibition is able to increase intracellular cAMP levels and, consecutively, downregulate the inflammatory response, by modulating the expression of *TNF-α*, IL-23, *IL-17*, and other pro-inflammatory cytokines. Moreover, this drug induces the production of anti-inflammatory cytokines, including IL-10 [50]. Preclinical studies revealed that apremilast alleviated the epidermal thickness and abnormal proliferation, and reduced the expression of *TNF-α*, HLA-DR, and ICAM-1 in the lesions. Apremilast is absorbed from the gut and is mainly metabolized by CYP450 3A4. On the other hand, this drug may cause adverse reactions, such as headache, abdominal pain, depression, weight loss, nausea, diarrhea, vomiting, and nasopharyngitis.

Long-term administration of apremilast revealed a different degree of response to the treatment, suggesting the possibility to plan personalized therapy. In this regard, a whole-genome SNP coverage analysis was performed to assess the association between genetic variants and apremilast therapy outcome. This investigation identified four SNPs (rs1143633, C/T; rs20541, G/A; rs2201841, A/G/T; rs1800629, A/G) located within different genes (*IL-1β*, *IL-4*, *IL-23R,* and *TNF-α*) involved in the inflammatory and immune response (Table 3). In particular, patients with the minor allele showed a better outcome to apremilast administration [51]. It is important to remark that these associations are preliminary results and further studies are terribly necessary. Indeed, the impact of pharmacogenetics on the response to apremilast is a field under active investigation.

#### 3.2.2. Fumaric Acid Esters

Fumaric acid esters (FAEs) are lipophilic ester derivatives of fumaric acid, and are recommended by the European guidelines for the management of moderate-to-severe plaque Ps. Dimethyl fumarate (DMF) is an orally administered FAE, indicated for the treatment of plaque Ps in adults. DMF acts as an immunomodulatory agent, which inhibits NF-κB translocation [52]. In addition, this small molecule induces the reduction in inflammatory cytokine production, blocks the proliferation of keratinocytes, alters the expression of adhesion molecules, and decreases inflammatory infiltrate within psoriatic plaques [52]. This drug showed a good efficacy and tolerable safety profile. The most common adverse events (flushing and gastrointestinal disorders) occurred mainly during the first few weeks of treatment.

#### 3.2.3. JAK Inhibitor

The novel class of drugs investigated for the treatment of different immune-mediated and inflammatory disorders are the Janus kinase (JAK) inhibitors. The JAK family includes JAK1, JAK2, JAK3, and TYK2 that transduce cytokine-mediated signals via the JAK-STAT pathway. This pathway plays an important role in the modulation of cytokines directly involved in the inflammation. Tofacitinib (TOF) is an oral JAK1 and JAK3 inhibitor that was firstly approved for the treatment of rheumatoid arthritis (November 2012). Afterwards, the FDA allowed the use of TOF to treat PsA (December 2017). Although TOF was effective for the treatment of moderate-to-severe plaque Ps, the FDA declined its approval because of the high incidence of ADRs [53]. The common side effects are upper respiratory tract infections, headache, diarrhea, and cold symptoms, such as a sore throat, or runny or stuffy nose. On the other hand, different research studies demonstrated that TOF affects the pathophysiological processes involved in Ps etiopathogenesis. In fact, this small molecule influences the dysregulation of keratinocytes, induces the reduction in inflammatory infiltrates, and normalizes the IL-23/Th17 axis. It is important to highlight that pharmacogenetic data concerning the differential response and ADRs risk in the treatment with TOF are not available.

## 4. Conclusions

To date, a wide range of treatments are available for the management of Ps. The treatment choice depends on the disease severity and the response degree of patients to previous therapy. The selection of the therapy also depends on whether the patient suffers from comorbidities (such as PsA), on the speed of drug effect, its sustainability, and its safety profile. Clinicians often need to try different drugs or a combination of them before, to identify the optimal therapeutic approach for each patient, which can be very challenging in some cases. In this scenario, clinicians could consider the possible effects of the patient’s genomic profile on the therapy outcome. As a matter of the fact, patients respond differently to the administrated therapy [54]. In this regard, pharmacogenomics can represent a useful tool to maximize the effectiveness of treatments, and reduce their adverse effects. In fact, the research of genetic variants located in genes encoding for drug-metabolizing enzymes, drug receptors, and drug transporters have been associated with the possibility of predicting an individual response to drugs. The chance of proposing pharmacogenomic tests becomes even more important to promote safer and more effective use of drugs, given the possibility to identify patients for which a specific drug can be dangerous, rather than therapeutic. In this context, an excellent example of the application of pharmacogenomic tests is the genetic screening for the HLA-B*57:01 allele in HIV-infected patients, before the administration of abacavir, which has been mandatory since July 2008.

Concerning the drugs in use for the treatment of Ps, the scenario of pharmacogenetic approaches requires further investigation, in order to establish a set of biomarkers to be introduced into clinical practice. So far, the pharmacogenomic studies performed on genes involved in the metabolism of biological drugs and small molecules have provided conflicting results. Moreover, the association between possible biomarkers and biologics/SMIs varies between populations, suggesting the existence of population-specific pharmacogenomic biomarkers. In addition, these data should be adjusted for the different prevalence of polymorphisms among populations.

Several studies reported that SNPs located in *HLA-A*, *TNF*, *ILs*, *HLA-B,* and *HLA-C* are associated with the response to anti-TNF drugs. Moreover, *ILs* and *TNFAIP3* genes may be involved in the development of toxicity and paradoxical Ps in patients treated with anti-TNF drugs. Pharmacogenomic analyses demonstrated that psoriatic patients showed a different response to the treatment with UTK. On the other hand, pharmacogenomic details on BDL, GSL, TDK, RSK, and SMIs are lacking. To date, new potential SMIs (amygdalin analog, protein kinase C inhibitors, fumaric acid esters) are under investigation for the treatment of Ps, for which the development of personalized medicine protocols requires further research studies.

In conclusion, the application of personalized medicine protocols into clinical practice will represent an excellent tool to manage the disease and identify the optimal therapy in relation to the patient’s genomic profile, thereby contributing to preventing ADRs and reducing the economic burden of Ps.

## Figures and Tables

**Table 1:** Genes and genetic variants potentially involved in the response to anti-TNF therapy.

Therapy	Gene	Locus	Genetic Variants
Anti-TNF drugs	*TNF-α*	6p21.33	rs1800629 A/G rs361525 G/A rs1799724 C/T rs1799964 T/C
	*TNFAIP3*	6q23.3	rs610604 G/A/C/T rs6920220 G/A
	*IL-12B*	5q33.3	rs2546890 A/G
	*IL-1B*	2q14.1	rs1143623 C/A/G rs1143627 G/A
	*IL-6*	7p15.3	rs1800795 C/G/T
	*IL-17A*	6p12.2	rs763780 C/T
	*IL-17-RA*	22q11.1	rs4819554 G/A/C
	*IL-23R*	1p31.3	rs11209026 A/G
	*HLA-B*	6p21	rs13437088 C/T
	*HLA-C*	6p21	rs1048554 G/A/C HLA-Cw*06
	*AKAP13*	15q25.3	rs28461892 C/A
	*HNRNPKP3*	11p12	rs11037360 G/A rs7481533 T/A/C/G rs11037342 A/C/T rs145304743 G/A/T/C rs1845821 A/G/T
	*SUPT3H*	6p21.1	rs9472377 A/G
	*NPFFR2*	4q13.3	rs13139992 G/A rs77656238 C/A/T
	*CDH12*	5p14.3	rs1487419 G/A rs77497886 G/T rs80063785 A/G

**Table 2:** Genes and genetic variants potentially involved in the response to cytokine inhibitors.

Therapy	Gene	Locus	Genetic Variants
Cytokine inhibitors	*IL-17*	6p12.2	rs763780 C/T rs3748067 C/T rs2275913 C/G/T rs3819025 A/G rs7747909 A/G rs8193037 A/C/T
	*IL-17RA*	22q11.1	rs4819958 G/A
	*HLA-C*	6p21	HLA-C*06:02
	*MICB-DT*	6p21.33	rs9267325 G/C
	*DDX58*	9p21.1	rs34085293 T/G
	*TYK2*	19p13.2	rs2304255 C/T
	*IL-12B*	5q33.3	rs2546890 A/G rs3213094 C/G/T
	*IL-1B*	2q14.1	rs1143623 C/G/T rs1143627 A/G
	*TNFAIP3*	6q23.3	rs610604 G/A/C/T
	*TLR2*	4q31.	rs4696480 A/T rs11938228 A/G/T
	*TLR5*	1q41	rs5744174 A/G
	*TLR9*	3p21.2	rs352139 G/A/T/C
	*PGLYRP4*	1q21.3	rs2916205 C/T
	*FBXL19*	16p11.2	rs10782001 G/A/C
	*CARD14*	17q25.3	rs11652075 C/T

**Table 3:** Genes and genetic variants potentially involved in the response to apremilast.

Therapy	Gene	Locus	Genetic Variants
Apremilast	*IL-1B*	2q14.	rs1143633 C/T
	*IL-4*	5q31.1	rs20541 G/A
	*IL-23R*	1p31.3	rs2201841 A/G/T
	*TNF-α*	6p21.33	rs1800629 A/G
